# Depletion of transmembrane mucin 4 (Muc4) alters intestinal homeostasis in a genetically engineered mouse model of colorectal cancer

**DOI:** 10.18632/aging.203935

**Published:** 2022-03-07

**Authors:** Ramesh Pothuraju, Priya Pai, Sanjib Chaudhary, Jawed A. Siddiqui, Jesse L. Cox, Sukhwinder Kaur, Satyanarayana Rachagani, Hemant K. Roy, Michael Bouvet, Surinder K. Batra

**Affiliations:** 1 Department of Biochemistry and Molecular Biology, University of Nebraska Medical Center, Omaha, NE 68198, USA; 2 Department of Pathology and Microbiology, University of Nebraska Medical Center, Omaha, NE 68198, USA; 3 Department of Medicine, Baylor College of Medicine, Houston, TX 77030, USA; 4 Division of Surgical Oncology, Department of Surgery, University of California San Diego, La Jolla, CA 92093, USA; 5 VA San Diego Healthcare System, San Diego, CA 92161, USA; 6 Fred and Pamela Buffett Cancer Center, University of Nebraska Medical Center, Omaha, NE 68198, USA; 7 Eppley Institute for Research in Cancer and Allied Diseases, University of Nebraska Medical Center, Omaha, NE 68198, USA

**Keywords:** mucin, MUC4, intestinal homeostasis, colorectal cancer

## Abstract

Mucins are components of the mucus layer overlying the intestinal epithelial cells, which maintains physiological homeostasis. Altered mucin expression is associated with disease progression. Expression of MUC4 decreases in colorectal cancer (CRC); however, its functional role and implications in the intestinal pathology in CRC are not studied well. Therefore, we generated a genetically engineered Muc4 knockout (Muc4^-/-^) CRC mouse model by crossing with Muc4^-/-^ and *Apc^flox/flox^* mice in the presence of colon-specific inducible Cre. We observed that deficiency of Muc4 results in an increased number of macroscopic tumors in the colon and rectal region and leads to poor survival. Further, the absence of Muc4 was associated with goblet cell dysfunction where the expression of intestinal homeostasis molecules (Muc2 and Fam3D) was downregulated. Next, we also observed that loss of Muc4 showed reduced thickness of mucus layer, leading to infiltration of bacteria, reduction in anti-microbial peptides, and upregulation of pro-inflammatory cytokines. Further, *Apc* gene mutation results in activation of the Wnt/β-catenin signaling pathway that corroborated with an increased nuclear accumulation of β-catenin and activation of its target genes: cyclin D1 and c-Myc in Muc4^-/-^ mice was observed. We conclude that the presence of Muc4 is essential for intestinal homeostasis, reduces tumor burden, and improves overall survival.

## INTRODUCTION

Colorectal cancer (CRC) occurs through a multi-step process (polyp-adenoma-carcinoma) in which several mutations are associated with disease progression [1, 2]. The most frequent mutations (40-70%) occur in the adenomatous polyposis coli (APC) gene, a component of Wnt/β-catenin signaling [2, 3]. CRC is the third leading cause of cancer-related deaths in the United States of America and will account for approximately 1,49,500 new cases and 52,980 deaths in 2021 [4]. As a result, studies have been conducted to understand CRC progression and various mouse models to identify potential treatments [5–7]. However, xenograft and chemically induced mouse models fail to recapitulate human CRC [7]. For instance, CRC induced by carcinogen affects several organs and also depends on route, dosage, and treatment duration [8]. Thus, genetically engineered mouse (GEM) models have great utility in studying CRC progression and can be used for evaluating pre-clinical therapeutic effectiveness. For instance, the sporadic CRC animal model has conditional mutations in the *Apc* gene that develops tumors in the colon and rectal region by using the Cre-Lox system.

Intestinal epithelial cells in the gut maintain homeostasis by preventing the entry of pathogens [9]. Such goblet epithelial cells produce several anti-microbial peptides and mucins, which are part of the mucus layer in the intestine [10]. Mucins are high-molecular-weight *O*-linked glycoproteins, and their altered expression or aberrant glycosylation is associated with many cancers [11, 12]. For example, the transmembrane mucin MUC4 is overexpressed in pancreatic, ovarian, and breast cancers and is involved in metastasis [13–16]. In contrast, expression of MUC4 was significantly downregulated during adenoma and adenocarcinoma progression in CRC tissues as evident from human microarray analyses [17]. Initial identification of MUC4 expression was done in CRC tumors by Ogata and co-workers [18]. The study results showed that four CRC tumors had reduced expression of MUC4, and the rest of the tumors either had higher expression or expression similar to normal tissue [18]. In another study, hyperplastic polyps had a reduction in MUC4 expression, while tubular adenoma samples showed normal levels of MUC4 in the same study [19]. Similarly, non-mucinous tumors, which were 66%, also had low to moderate levels of MUC4, while 34% of CRC patients had upregulation of MUC4 [20]. Thus, several studies suggested that MUC4 expression is lost or reduced in CRC [18, 21]. Despite loss or reduction of MUC4 expression in the majority (75%) of CRC patient tumors, a small subset (25%) of CRC patients had high expression of MUC4, particularly in stages I and II of the disease [22, 23]. Contrary, the pro-tumorigenic role of MUC4 has observed in chemical-induced colitis and CRC [24] models and hypothesized that the tumorigenic potential of MUC4 is due to truncated glycan epitopes present on it and altered binding affinity of MUC4 antibodies [25]. Thus, the role of MUC4 in homeostasis and disease pathology in the colon remains debatable. Therefore, to understand the functional significance of MUC4 in intestinal homeostasis and CRC progression, we developed a GEM model by crossing mice carrying a conditional mutation of *Apc* gene with colon-specific caudal type homeobox transcription factor 2 (Cdx2)-Cre fused with estrogen receptor.

## RESULTS

### Low MUC4 expression is associated with poor survival in CRC patients

**Figure 1:**
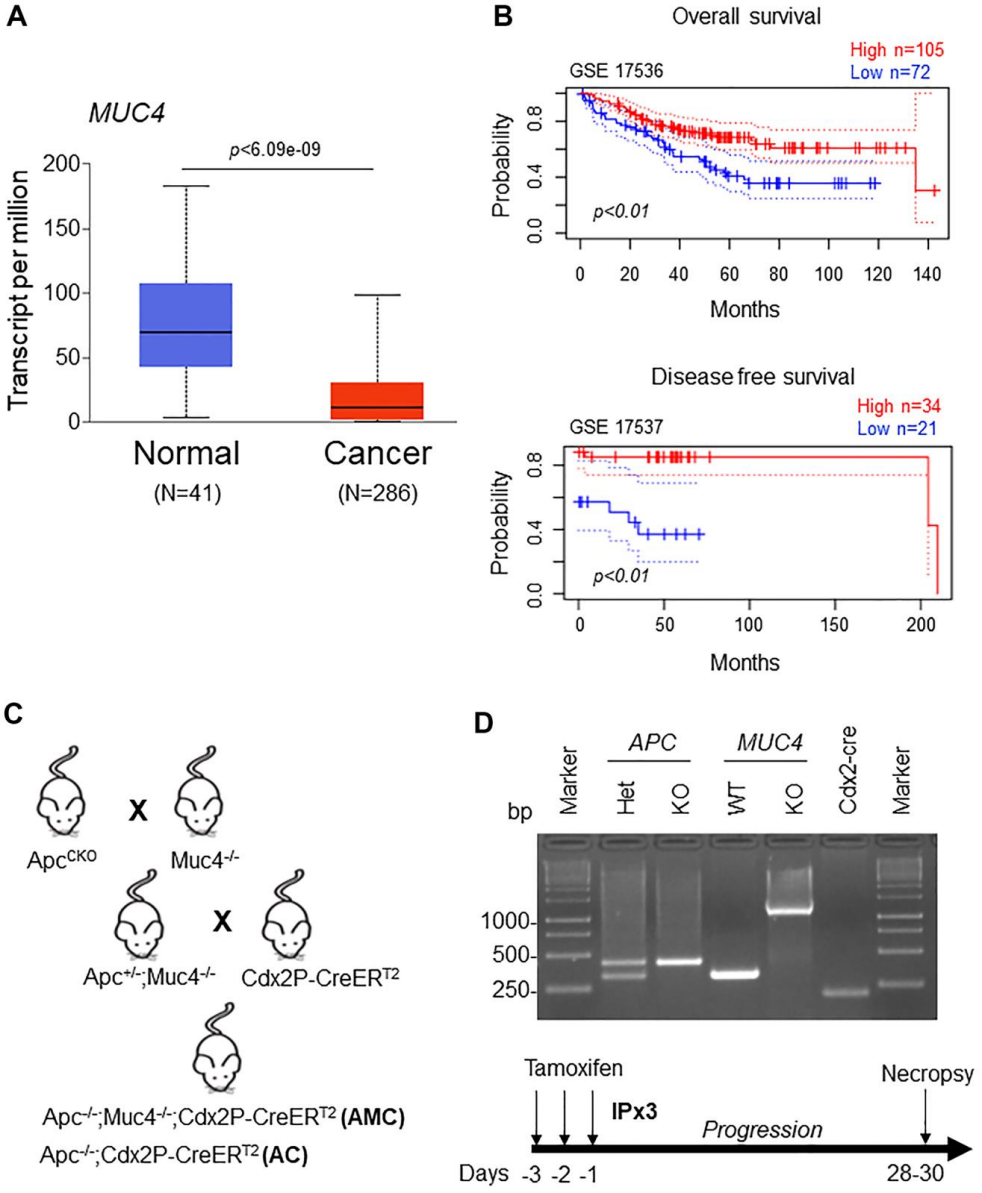
**Low MUC4 expression is associated with poor survival in CRC patients.** (**A**) A significant (*p* < 6.09e-09) upregulation of MUC4 in normal (N=41) and downregulation in CRC patients (N=286) was analyzed in the TCGA-COAD dataset. (**B**) Increased expression of MUC4 is associated with better overall and disease-free survival in CRC patients’ data sets (GSE 17536 and GSE 17537). (**C**) Breeding strategy for the generation of a genetically engineered mouse model for Muc4^-/-^ by crossing with Apc^flox/flox^ mice. First-generation of double knockout (Apc^-/-^;Muc4^-/-^) animals were further crossed with inducible colon-specific Cre (Cdx2P-Cre ER^T2^) mice to get final cross (Apc^-/-^;Muc4^-/-^;Cdx2P-CreER^T2^, AMC) and its littermate controls (Apc^-/-^;Cdx2P-CreER^T2^, AC). (**D**) PCR products of *Apc* and *Muc4* and *Cdx2-cre* animals of genomic DNA. Below, induction of Cre recombination by tamoxifen administration (75 mg/kg body weight, 3x) via intraperitoneally.

Our analysis showed a significant (*p*<0.05) down-regulation of *MUC4* expression in CRC patients compared to normal individuals by using The Cancer Genome Atlas (TCGA-COAD) database (containing 286 colon cancer and 41 normal samples) (Figure 1A). In addition, we also analyzed *MUC4* expression in two datasets (GEO accession: GSE17536, *p*<0.01 and GSE17537, *p*<0.01) and observed that patients with high *MUC4* expression showed a significant improvement in overall and disease-free survival (Figure 1B), suggesting a protective role of MUC4 in CRC.

### Muc4 deletion drives colorectal tumors with high-grade dysplasia

**Figure 2:**
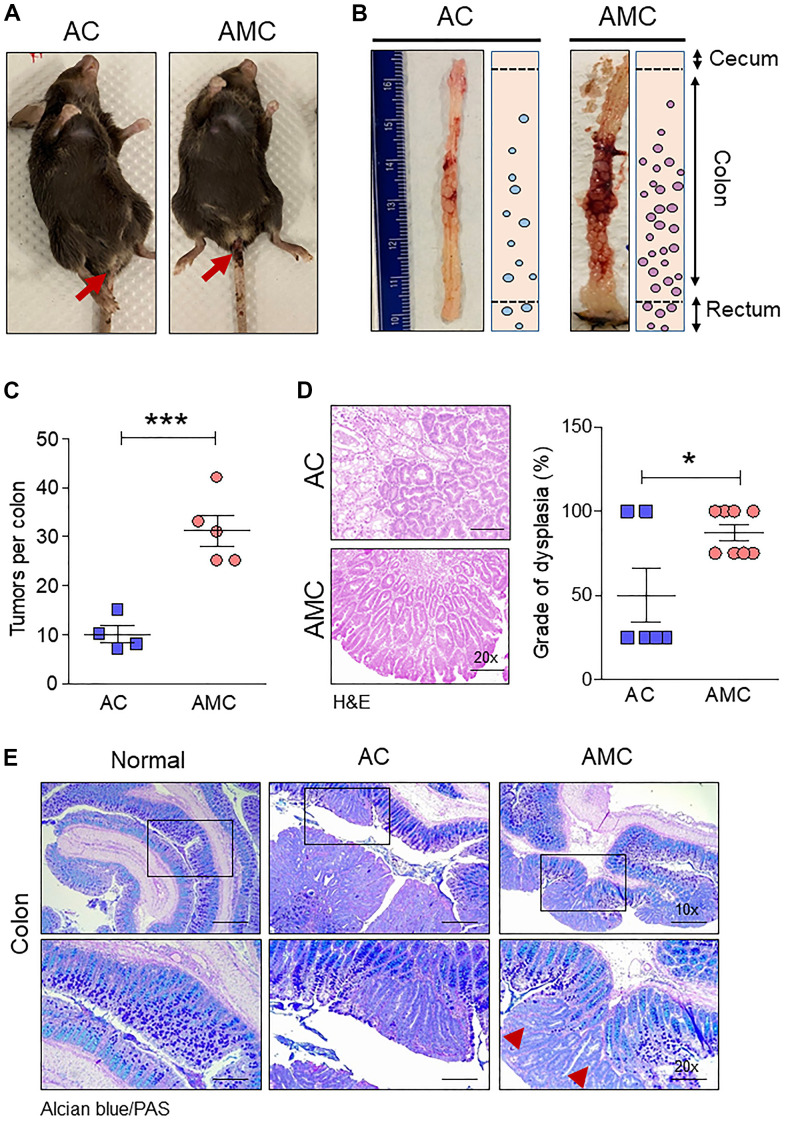
**Muc4 deletion drives colorectal tumors with high-grade dysplasia and goblet cell dysfunction.** (**A**) Representative images of AC and AMC animals had typical rectal bleeding. (**B**, **C**) Muc4^-/-^ mice in the AMC group showed an increase in the number of macroscopic polyps in the colon and rectal region. n =4-5 per group. (**D**) H&E staining of AMC animals had a higher grade of dysplasia. n = 6-8 per group. (**E**) Representative images of double staining of Alcian blue and PAS. n = 6 (AC and AMC) and n=3 for normal group. Red arrowhead indicates loss of mucins expression in the goblet cells in AMC mice. *p < 0.05, ***p < 0.001.

To understand the role of transmembrane mucin Muc4 in CRC initiation and progression, we generated Apc^-/-^;Muc4^-/-^;Cdx2P-creERT^2^ (referred to AMC) and its contemporary littermate control Apc^-/-^;Cdx2P-creERT^2^ (AC) mice (Figure 1C). After confirming genotype, cre-recombination was carried out by administering tamoxifen intraperitoneally (Figure 1D), and mice from both groups were euthanized based on the incidence of symptoms (rectal bleeding, kyphosis, and lethargy) (Figure 2A). AMC animals displayed an increased number of macroscopic polyps in the large intestine (Figure 2B, 2C). In addition, histological examination of colorectal tissue of AMC mice revealed that all adenomas were high-grade dysplasia without reaching muscularis layer of the colon (Figure 2D).

### Loss of Muc4 shows goblet cell dysfunction

**Figure 3:**
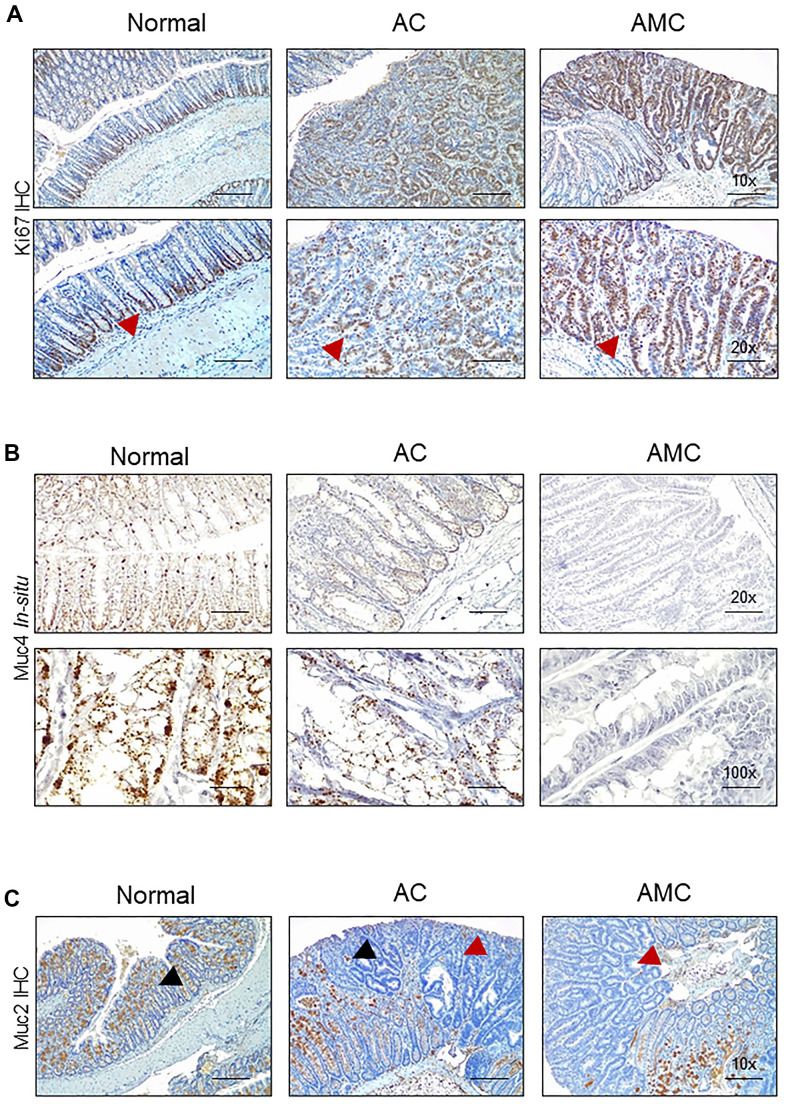
**Absence of Muc4 alters other mucins expression.** (**A**) Immunohistochemistry staining of Ki67 in the colon of normal, AC, and AMC mice. Red arrowhead indicates Ki67 staining restricts to crypts in normal animals, whereas AMC mice showed higher cell proliferation (more Ki-67 staining), which might be attributable to colonic crypt hyperplasia. (**B**) Staining of Muc4 was observed in normal, AC mice, whereas the complete absence of Muc4 expression in AMC animals was performed by *in-situ* hybridization. (**C**) Immunohistochemistry staining of Muc2 (Black arrowhead: positive staining and Red arrowhead: absence of staining in the tumors). n = 6 (AC and AMC) and n=3 for normal group.

Next, we characterized goblet cell function in all animal groups by staining Swiss rolled colon tissues with Alcian blue (stains acidic mucins) and Periodic acid–Schiff (PAS, neutral mucins). We observed that in both AMC and AC mice, there was a complete absence or loss of staining in the goblet cells of colon adenoma (Figure 2E), suggesting that disruption of goblet cell function alters the mucin production. There was an increase in the crypt length of AMC mice associated with strong Ki67-positive staining in the proliferating epithelial cells in the colon with a disorganized pattern. In contrast, normal animals showed Ki67 staining mostly restricted at the bottom of the crypt, absent in the epithelial cells towards the top of the crypt (Figure 3A).

### Absence of Muc4 alters other mucins expression

The expression of secreted mucin Muc2, which is a major component of the mucus layer, and the membrane-bound mucin Muc13, Muc16, and another secretory mucin Muc5ac (has a role in tumorigenesis, metastasis, and drug resistance) was examined. The complete loss or deletion of Muc4 in AMC mice (Figure 3B) was associated with a significant loss of Muc2 in the colon adenoma compared to normal mice (Figure 3C). However, the expression of other mucin Muc13 was up-regulated, while no changes in Muc16 and Muc5ac at transcript levels in AMC compared to AC mice. (Supplementary Figure 1A).

### Muc4 deletion results in defective mucus barrier function and reduced intestinal homeostatic molecules

**Figure 4:**
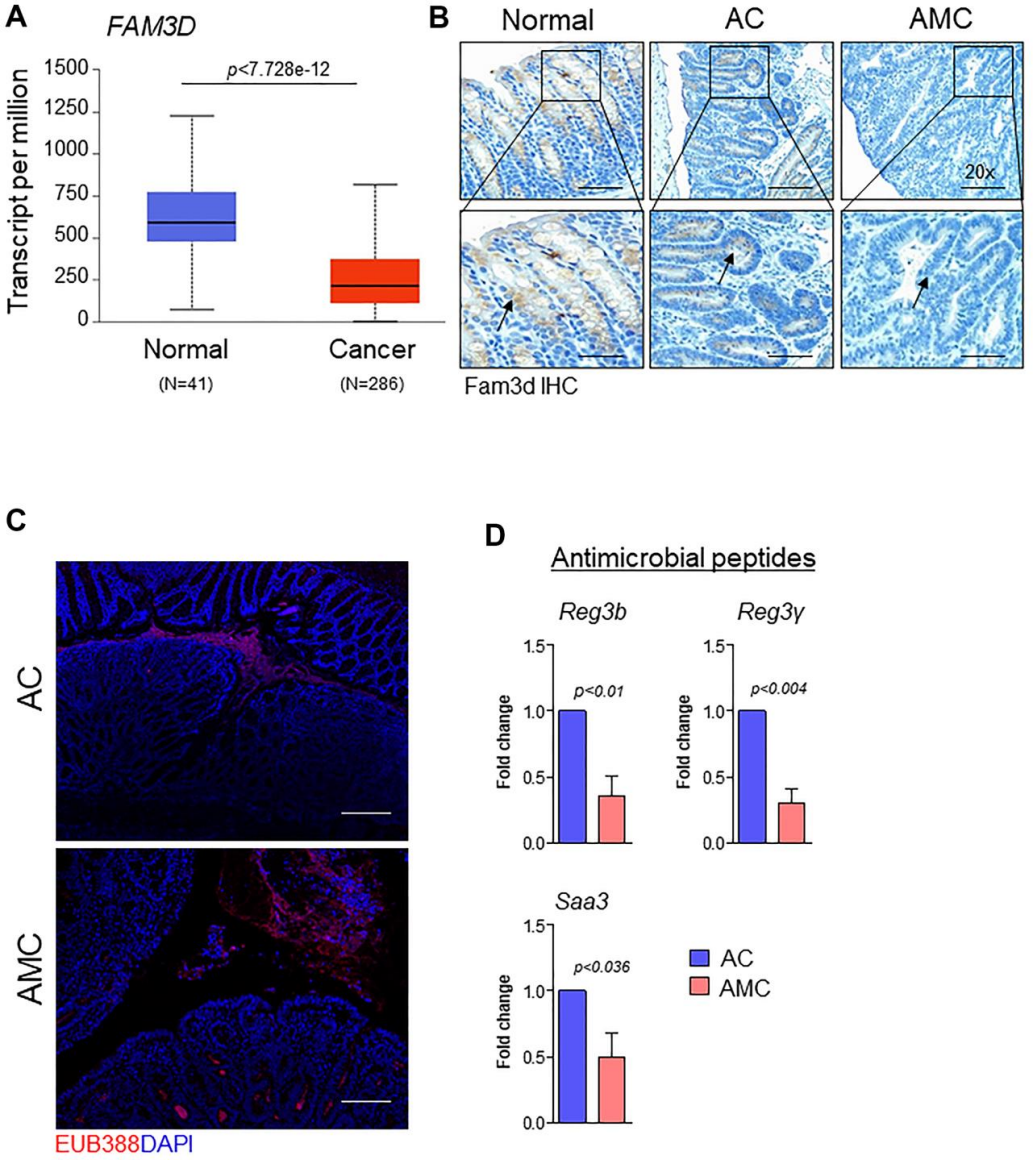
**Muc4 deletion results in defective mucus barrier function and reduced intestinal homeostasis molecules.** (**A**) TCGA-COAD dataset showed down-regulation of FAM3D in CRC patients (N = 286) compared with healthy controls (N = 41). (**B**) Immunohistochemistry analysis of Fam3d expression in the colon of normal, AC, and AMC mice. n = 6 (AC and AMC) and n=3 for normal group. (**C**) Representative images of immunofluorescent staining of bacteria (red color) done by fluorescence *in-situ* hybridization using EUB338-Cy3 probe. n = 3 per group. (**D**) mRNA expression levels of antimicrobial peptides (*Reg3b*, *Reg3γ,* and *Saa3*) in the colons of AC and AMC mice were measured by real-time PCR. n = 3-4 per group.

Like Muc2, the gut secreted protein Fam3d is essential for colon homeostasis [26]. Human FAM3 consists of four gene families: FAM3A, FAM3B, FAM3C, and FAM3D [26]. Among all FAM3 family members, the expression of *FAM3D* is significantly downregulated in human CRC patients compared to normal individuals (Figure 4A and Supplementary Figure 1B). Therefore, we examined the expression of Fam3d and observed that Fam3d was expressed in Muc2 positive goblet cells in the normal mice, while its expression was greatly diminished or lost in the adenoma region of AMC mice (Figure 4B), suggesting that loss of both Muc2 and Fam3d reduces intestinal mucus layer thickness in AMC animals. Both Muc2 and Fam3d have been involved in preventing the entry of bacteria maintaining the colon homeostasis, as evidenced in several studies [26, 27]. Therefore, we analyzed the colon tissue sections for bacterial invasion by FISH using a general 16S rRNA probe (EUB338). Compared to the AC, AMC mice colon had reduced mucus layer thickness, resulting in more bacteria infiltration in the colonic epithelial tissues (Figure 4C). Further studies are necessary to understand the various individual bacterial populations in the AMC model to determine the role of Muc4 in altering the gut microbiota. Next, we determined the expression of intestinal homeostatic antimicrobial peptides (*Reg3b*, *Reg3γ,* and *Saa3*) in colon of AMC and AC mice. Interestingly, we observed that the absence of Muc4 resulted in significant down regulation of these antimicrobial peptides at mRNA levels (Figure 4D). The absence of Muc4 in AMC mice resulted in severe bleeding in the large intestine (Figure 2A, 2B). To examine whether the bleeding recruits any cytokines and inflammatory cells, we analyzed the expression of proinflammatory cytokines such as *Tnfa, IL-6, Cxcl2, Ccl2, Cxcl1 and IL-1* and observed that AMC animals showed up regulation of cytokines (Supplementary Figure 2A) whereas, no difference in the expression of F4/80 and CD3^+^ T cells staining in AMC and AC animals (Supplementary Figure 2B).

### Muc4 deletion results in up-regulation of β-catenin signaling

**Figure 5:**
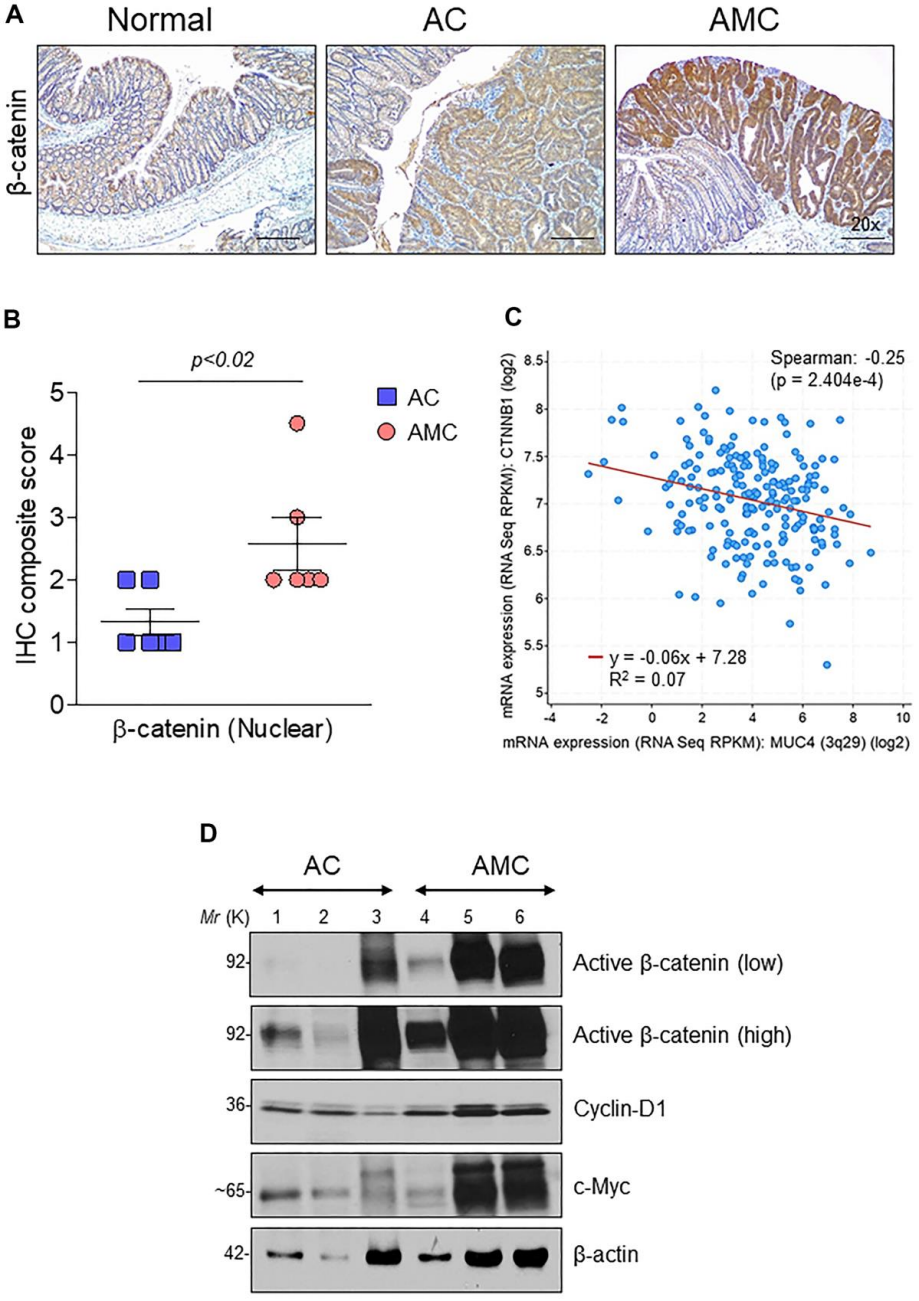
**Muc4 deletion results in up-regulation of β-catenin signaling.** (**A**) Immunohistochemical analysis of β-catenin in normal, AC and AMC animals. n = 6 (AC and AMC) and n=3 for normal group. (**B**) IHC composite score of nuclear β-catenin staining in AC and AMIC mice (**C**) MUC4 and CTNNB1 (β-catenin) correlated negatively in the TCGA-COAD dataset. (**D**) Immunoblot of active β-catenin and its target genes: cyclin-D1 and c-Myc expression in AC and AMC animals. n = 3 per group.

Wnt/β-catenin signaling plays an essential role in CRC progression. Around 90% of human CRC tumors contain mutations in the Wnt pathway, while 50-80% of CRC patients have an accumulation of β-catenin in the nucleus [28]. Therefore, we analyzed the expression of β-catenin in the nucleus by immunohistochemistry in both AMC and AC mice. Interestingly, AMC animals showed a significantly (*p*<0.02) higher nuclear accumulation of β-catenin compared to AC mice (Figure 5A, 5B). Similarly, we also identified a significant (*p*<2.404e-4) negative correlation between MUC4 and β-catenin in CRC patient dataset (Figure 5C). Further, mRNA and western blot analysis confirmed higher expression of β-catenin and its target molecules such as cyclin-D1, c-Myc, and CD44 in AMC mice (Supplementary Figure 3A and Figure 5D).

**Figure 6:**
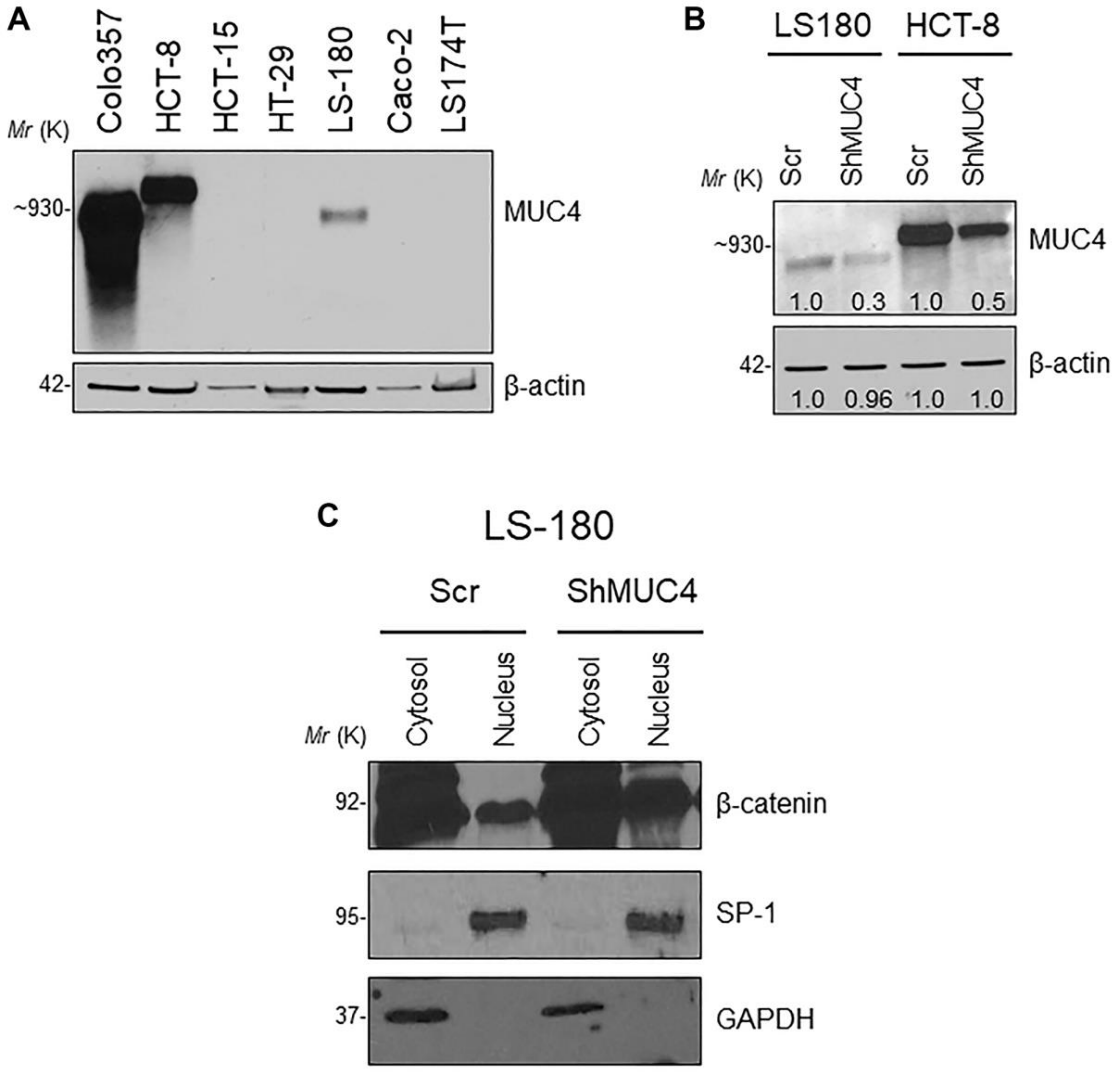
**Knockdown (KD) of MUC4 in CRC cell lines mediates β-catenin signaling.** (**A**) A panel of CRC cell lines was used and screened for MUC4 expression (Colo357 pancreatic cancer cell line used as a positive control). (**B**) Stable KD of MUC4 in both LS-180 and HCT-8 CRC cell lines. (**C**) Expression of nuclear β-catenin levels in a sub-cellular fraction of CRC cell line done by western blot.

Next, we assessed the relationship between the MUC4 and β-catenin in the human CRC cell line. First, we screened a panel of CRC cell lines for MUC4 expression (Figure 6A) and other mucins such as MUC5AC, MUC1, MUC13 (Supplementary Figure 3B). The MUC4 expressing LS-180 and HCT-8 CRC cell lines were selected and then performed stable knockdown of MUC4 in both CRC cell lines (Figure 6B). Knockdown (KD) of MUC4 increased the expression of β-catenin, cyclin-D1, and CD44 at the transcript level in LS-180 and HCT-8 cells (Supplementary Figure 3C). Our cellular fractionation study also revealed that the MUC4-KD in the LS-180 cell line resulted in increased localization of β-catenin in the nucleus compared to a cytosolic fraction (Figure 6C). These data suggest that the presence of Muc4 prevents nuclear translocation of β-catenin in both *in vitro* and *in vivo* studies; however, further studies are needed to prove the molecular mechanism by which Muc4 modulates the Wnt/β-catenin signaling pathway(s).

### Additional kras mutation aggravates Apc tumors and reduces survival

**Figure 7:**
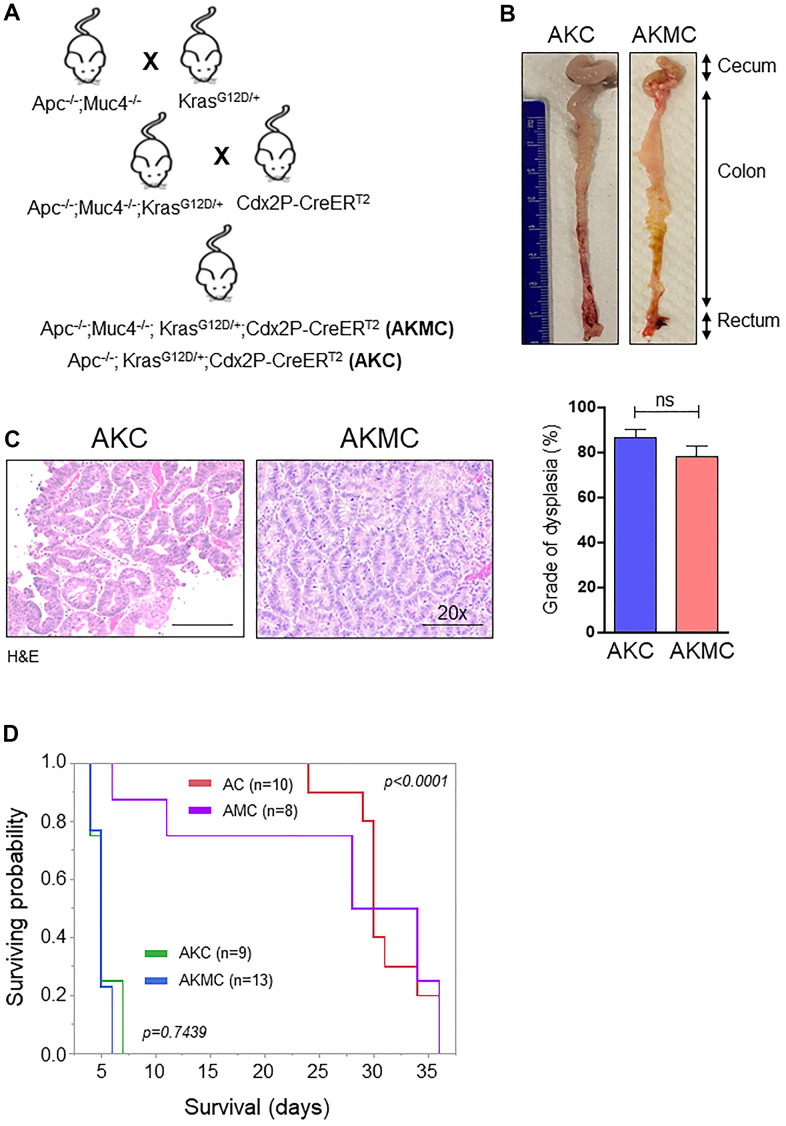
**Additional Kras mutation aggravates Apc tumors and reduces survival.** (**A**) Breeding strategy for generation of genetically engineered mice model for Muc4^-/-^ by crossing with Apc^flox/flox^ and Kras^G12D^ mutant mice. First-generation of Apc^-/-^;Muc4^-/-^;Kras^G12D/+^ animals were further crossed with inducible colon specific Cre (Cdx2P-Cre ER^T2^) mice to get final cross (Apc^-/-^;Muc4^-/-^; Kras^G12D/+^;Cdx2P-CreER^T2^, AKMC) and its littermate controls (Apc^-/-^;Kras^G12D/+^;Cdx2P-CreER^T2^, AKC). (**B**) Representative images of the colon in AKC and AKMC animals. (**C**) H&E images showed a grade of dysplasia in AKC and AKMC mice. n = 3 for AKC and n=6 for AKMC group. (**D**) Overall survival curves of different mouse models used in the present study. ns = non-significant.

Early CRC tumors typically possess 30-50% activation mutations in the *Kras* (45%) gene along with *Apc* mutations [3]. Hence, to understand the significance of *Kras* mutations in CRC, we crossed LSL-Kras mice [B6.129-Krastm4Tyj (01XJ6) from NCI Mouse Models of Human Cancers Consortium, Frederick, MD, USA] [29] with AMC and generated Apc^-/-^;Kras^G12D/+^;Muc4^-/-^; Cdx2P-creERT^2^ (referred as AKMC) and its littermate controls Apc^-/-^; Kras^G12D/+^;Cdx2P-creERT^2^ (AKC) (Figure 7A). The activation of cre-recombination was carried out as described in Figure 1D. Interestingly, additional kras mutation in AKMC mice did not observe any macroscopic tumors in the colon (Figure 7B). However, tumor histology of the colon reveals both AKMC and AKC animals had low-grade dysplastic (Figure 7C). Further, these animals had a shorter life span than AMC and AC mice (Figure 7D).

## DISCUSSION

Transmembrane mucin MUC4 is differentially expressed in various cancers. In early gastric cancer, expression of MUC4 was associated with lymphatic invasion, lymph node metastasis and predicted poor prognostic factors [30]. Further, the importance of MUC4 expression was identified in invasive ductal carcinoma (ICD) of the pancreas. The study concluded that in surgical resected IDC, MUC4 could serve as a valuable indicator to predict the outcome of patients [31]. Mechanistic studies suggest that the presence of MUC4 mediates pancreatic cancer cell invasion and metastasis by stabilizing fibroblast growth factor receptor 1 [32] and oncogenic signaling via interaction with HER3 [33]. Contrary, MUC4 has a tumor suppressor role in non-small-cell lung cancer by altering p53 expression [34]. Therefore, the present study focused on understanding the role of MUC4 in CRC progression and alteration of intestinal barrier function by developing a GEM model having an Apc mutation with or without Muc4 using colon-specific Cdx2-Cre mice. Previously, we showed that MUC4 expression is significantly downregulated in human CRC patient tissues compared to normal individuals [17]. Similarly, the TCGA-COAD database also showed that lowered expression of MUC4 in normal individuals is associated with poor survival. Therefore, we investigated the potential role of Muc4 in CRC by using a GEM model with an Apc mutational background.

In our study, loss of Muc4 in AMC mice resulted in severe rectal bleeding with more adenomas in the colon and rectal regions. We speculated that bleeding in AMC animals might be due to the infiltration of immune cells and the release of pro-inflammatory cytokines. Interestingly, we noticed high expression (without significant level) of inflammatory cytokines whereas infiltration of F4/80^+^ macrophages and CD3^+^ T cells was higher in both AMC and AC compared to normal animals, suggesting a low level of inflammation in the mice colons.

The intestinal mucus layer comprises different mucins and is a key barrier in the colon to prevent bacterial invasion into the colon epithelium [3, 35]. Our results demonstrate that combined loss of Apc and Muc4 in AMC animals impaired mucosal barrier function with reduced expression of mucins (confirmed by Alcian/PAS staining), especially Muc2. Secretory mucin Muc2 is abundant mucin in the intestinal mucus layer [27]. It is a large glycoprotein having *O*-linked glycans attached on its domains. The colon has two dramatic mucus layers: the outer (loosely attached) and the inner layer (tightly attached), which is devoid of bacteria [3, 36, 37]. The terminal sulfate and sialic acids on mucins are degraded by the microbial enzymes (sialidases and glycosulfatases) [38–40], resulting in the loss or reduction of mucin expression in the outer mucus layer of the large intestine to alter gut flora. In a study, Muc2^-/-^ mice displayed infiltration of bacteria into crypts due to intestinal barrier damage, which triggered an inflammation-mediated CRC [27], and its absence promotes CRC progression [41]. Thus, lack or loss of Muc2 expression in AMC mice was associated with a reduction in the mucus layer thickness and downregulation of antimicrobial peptides, which was associated with gut dysbiosis that resulted in infiltration of bacteria into the epithelium. Based on our findings, we conclude that the deletion of Muc4 may contribute to goblet cell dysfunction, resulting in alteration of mucin expression (Muc2), leading to dysbiosis. Along with MUC2, MUC13, MUC16, and MUC5AC have been implied in CRC progression, metastasis, and chemoresistance [42–44]. In the present study, we observed that Muc13 was upregulated in the absence of Muc4, suggesting its tumor-promoting role in CRC. Further studies on Muc4 and other mucus components responsible for the mucus layer formation and maintaining intestinal homeostasis are needed.

Like Muc2, a gut secreted protein, FAM3D is also essential for maintaining colon homeostasis [26]. Genetic deletion of Fam3D^-/-^ in mice showed decreased intestinal integrity, hyper-proliferation, and reduction in anti-microbial peptide production. Further, the absence of Fam3D was associated with altered gut microbiota [26]. Interestingly in our AMC animals, we observed that less expression of Fam3D compared to AC mice, which was corroborated in a human CRC patients’ dataset. Thus, we concluded that the decreased expression of Fam3D leads to more bacterial invasion into the intestinal epithelial cells of AMC animals. However, the relationship of mucins (Muc4 & Muc2) and Fam3D and their significance in facilitating microbiota composition need to be addressed in future studies.

Dysregulation of Wnt/β-catenin signaling is involved in several types of cancer, including CRC, to promote cell proliferation, invasion, and metastasis [45–47]. Mutation in the *APC* gene stabilizes cytoplasmic unphosphorylated β-catenin to enter the nucleus. It binds and activates transcription factors (T cell factor or lymphoid enhancer factors) to transcribe its target genes for CRC progression [44, 48, 49]. Previously, we have shown that Muc4 is negatively regulated through the Wnt/β-catenin signaling in CRC cell lines [50]. In the present study, we observed a higher nuclear accumulation of β-catenin in AMC mice colon tissues, resulting in upregulation of its target genes cyclin-D1 and c-Myc, which are essential for the proliferation of colonic epithelial cells. Further, to understand the relationship between Muc4 and β-catenin, we performed a stable knockdown of MUC4 in the human CRC cell line. Decreased expression of MUC4 results in more expression of β-catenin in the nuclear fraction. In contrast, Muc13 protects β-catenin degradation from glycogen synthase kinase 3β there by inducing nuclear translocation of β-catenin and activating the Wnt/β-catenin signaling pathway in a dextran sodium sulfate-induced CRC mice model [44]. Other studies have shown MUC1, and MUC4 have been to promote nuclear localization of β-catenin in gastrointestinal and colorectal cancers [44, 50, 51]. The presence of Muc4 might be associated with the β-catenin destruction complex components, which results inhibit the nuclear accumulation. However, the mechanism(s) by which Muc4 tethers the β-catenin in the cytosol needs to be studied.

Over the past decades, GEM models for CRC have been developed [49, 52–55]. The most used CRC mice model is Apc^Min/+^ mice; however, this model develops around 20-30 tumors mostly in the small intestine rather than the colon [56, 57]. Currently, several CRC mice models have been developed, which closely mimic human CRC, and these models form tumors mainly in the large intestine and occasionally in the small intestine [6, 7, 58, 59]. Here, we used Apc^flox/flox^ mice and observed tumors mostly in the large intestine; however, germline Apc mutations do not promote spontaneous Kras mutations [6, 60]. Around 50% of CRC patients have activating mutations in Kras [61]. Therefore, we introduced the activated *Kras* gene in our AMC and AC mice models and investigated whether the incorporated *Kras* gene would exacerbate invasive tumor progression and metastasis. We did not observe any macroscopic tumors in both AKMC and AKC mice, though histological analysis revealed more dysplasia without invasive lesions and no metastasis, which is corroborated with other studies [5]. Moreover, these mice had lower survival due to kyphosis, weight loss, rectal prolapse, and bleeding, indicating early euthanasia compared to AMC and AC mouse models, resultant in Kras activation [62]. Contrasting evidence with the addition of the *Kras* allele is reported to promote invasive tumors and metastasis [6, 63–65]. However, such discrepancies in the phenotype might be associated with invasive techniques used to activate Cre. For instance, localized adenoviral infection of the colon by incision near the anus [6] or introducing Cre-expressing virus through enema [59, 66], allowing these animals to survive for the long-term. Therefore, localized activation of Cre in AKMC mice will shed light on the importance of Muc4 in CRC progression and metastasis potential as these mice will survive for the long-term.

In conclusion, our study suggests that Muc4 has a protective role in CRC progression in an Apc mutant GEM mice model. Muc4 maintains the intestinal homeostasis by upregulation of Muc2 and Fam3D (guardians of the gut) and downregulation of cancer-promoting mucin (Muc13). Additionally, presence of Muc4 prevents the invasion of microbiota and reduction of proinflammatory cytokines and decrease in epithelial cell proliferation by inhibiting β-catenin, c-Myc and CD44 expression. Additional studies are needed to understand the role of Muc4 in conditional KO mouse models and various sub-types of CRC.

## MATERIALS AND METHODS

### Animals and handling

All the animals used in the present study were maintained in accordance with guidelines and protocols approved by the Institutional Animal Care and Use Committees (IACUC) of the University of Nebraska Medical Center, Omaha, NE, USA. Apc conditional knockout (Apc CKO, B6.Cg-Apctm2Rak/Nci) and Cdx2P-CreER^T2^ (Stock No: 022390) mice were obtained from the Mouse Model of Human Cancers Consortium (NCI-Frederick, MD, USA) and Jackson Laboratory (Bar Harbor, ME, USA), respectively. *Muc4* knockout (*Muc4*^-/-^) mice generated in-house has been previously described [24]. To generate CRC mice model with or without *Muc4^-/-^,* first we crossed mice carrying *lox*P-flanked *Apc* alleles (*Apc^flox/flox^)* with *Muc4^-/-^* to get AM mice. Second, F1 generation of AM animals were then crossed with Cdx2P-CreER^T2^ mice to generate AMC and its compound littermate’s AC animals. After confirming the genotype for all the mice, colon-specific activation of Cre recombinase was carried out by administering tamoxifen (Sigma-Aldrich, St Louis, MO, USA) via intraperitoneal injection (75 mg/kg body weight) for three consecutive days [29]. All mice (both male and female) used in the present study kept under a 12 h light/dark cycle, and fed a standard chow diet, and water *ad libitum*. The mice were euthanized by CO2 asphyxiation followed by cervical dislocation when they have blood in the stools, hunched appearance, and rectal prolapse.

The primers used for genotyping were as follows: ApcFP 5’-GAGAAACCCTGTCTCGAAAAAA-3’, ApcRP 5’- AGTGCTGTTTCTATGAGTCAA C-3’ (Wild type=320 bp, Loxp=430 bp); Muc4FP 5’-GCCTTCTATGAAAGGTTGGGCTTCG-3’, Muc4RP 5’-TCCCTTCCGGTGAAGCCTCT-3’ (Wild type=331 bp, Mutant=1319 bp); Cdx-creFP 5’-CATGGTGAGGTCTGCTGATG-3’, Cdx-creRP 5’-CATGTCCATCAGGTTCTTGC-3’ (transgene ~200 bp).

### Antibodies and other reagents

Rabbit anti-mouse mucin 2 (MUC2) antibody (H-300), mouse anti-F4/80 (sc-20047), and mouse anti-CD3 (sc-377009) were obtained from Santa Cruz (Dalla, TX, USA). MUC4 (8G7) in-house generated antibody. MUC5AC antibody (CLH2, MAB2011) from Millipore. Human anti-β-catenin (C2206) and β-actin (A1978) from Sigma-Aldrich (St. Louis, MO, USA). The goat anti-mouse FAM3D antibody (AF3021) was purchased from R&D Systems (Minneapolis, MN, USA). Non-phospho (Active) β-catenin (D2U8Y), β-catenin (D10A8), SP1 (5931), Cyclin-D1 (92G2) and c-Myc (D3N8F) obtained from Cell Signaling Technology (Danvers, MA, USA). Rabbit anti-Ki-67 (ab15580), MUC1 (ab70475) and MUC13 (ab65109) from Abcam (Cambridge, MA, USA). 4′,6-diamidino-2-phenylindole (DAPI Fluoromount-G®) from Southern Biotech (Birmingham, AL, USA). was obtained from Sigma-Aldrich (St. Louis, MO, USA).

### Cell lines and culture conditions

HCT-8, LS180, SW480 and HCT-15 cell lines were obtained from American Type Culture Collection and cultured in DMEM supplemented with 10% fetal bovine serum (FBS) and antibiotics (100 U/ml penicillin and 0.1 mg/ml streptomycin) at 37° C with 5% CO2 in a humidified atmosphere.

### MUC4 silencing by shRNA transfection

The stable knockdown (KD) of MUC4 was done in the LS-180 CRC cell line by a small hairpin RNA construct (pSUPER-Retro-shMUC4). The transfection of both scrambled and MUC4-KD constructs was followed as mentioned previously [43, 50].

### Tissue processing and histological analysis of mucins

Colon tissue was harvested for AC and AMC mice between 25-35 and 5-25 days, respectively, after tamoxifen induction. The colon was open longitudinally, and fecal material was gently removed. After washing with phosphate-buffered saline (PBS), a Swiss roll of the colon was made. For histology, tissues were fixed with 10% neutral buffered formalin prior to paraffin embedding. Both AC and AMC colon tissues were stained with hematoxylin and eosin, and pictures were taken with Leica ICC50 E microscope (Buffalo Grove, IL). Histological examination of grade of dysplasia was determined by the pathologist. To stain mucins, deparaffinized mouse colon tissues were immersed in Alcian blue followed by Periodic acid-Schiff (PAS) staining.

### *In situ* hybridization

Single-color mouse-specific 20-paired double-Z oligonucleotide probes were used for *in situ* hybridizations of MUC4 (NM_080457.3, target region 7065 - 7996) (Advanced Cell Diagnostics, Newark, CA, USA). Briefly, tissue sections were baked for 1 hr at 60° C for de-paraffinization, followed by protease treatment for 30 min at 40° C. Pre-heated target probes (MUC4, positive control, and negative control, RNAscope® 2.5 HD Assay- BROWN, RNAscope®) were hybridized for 2 hr at 40° C, followed by a series of signal amplification and washing steps using the HybEZ Hybridization System. Hybridization signals of probes were detected by sequential chromogenic reactions using brown chromogens, and images were captured with the Leica ICC50 E microscope as described earlier [67].

### Immunohistochemistry

The immunohistochemistry procedure has been described previously [43]. Both AC and AMC mice colon tissue sections were baked in an oven at 58° C overnight and deparaffinized in xylene and hydrated through different graded alcohols. Epitope retrieval was done by using 0.01M citrate buffer for 15 min, followed by quenching endogenous peroxidase activity for 1 hr in the dark. The sections were then blocked with 2.5% horse serum and then incubated with respective primary antibodies at 4° C overnight. The next day after washing, slides were probed with secondary HRP labelled universal anti-mouse/rabbit IgG for 1 hr and then added DAB substrate kit (Vector Laboratories, Burlingame, CA, USA) according to the manufacturer. Finally, nuclei were counterstained with hematoxylin, and intensity scoring was done by a pathologist. The composite score was calculated as mentioned previously [43, 68].

### Fluorescence *in situ* hybridization (FISH)

The FISH procedure was performed according to Cassmann et al., 2016 with slight modifications [69]. Briefly, tissues were dewaxed in xylene (3x10 min) and washed in 100% alcohol (2x5 min), 95% (5 min), and 70% (5 min). After ethanol wash, slides were further rinsed with deionized water and air-dried. Tissue sections were incubated with 100 ng/ul of 5’-Cy3 labelled universal DNA-probe mix EUB338 (5′-GCTGCCTCCCGTAGGAGT-3′) or with a non-specific negative probe NON338 (5′-ACTCCTACGGGAGGCAGC-3′, both purchased from Eurofins MWG Operon, Louisville, KY) in a hybridization buffer (20 mM Tris-HCl, pH 7.4; 0.9 M NaCl; 0.1% sodium dodecyl sulfate) at 37° C overnight. The next day, tissues were rinsed with wash buffer (20 mM Tris-HCl, pH 7.4; 0.9 M NaCl) at 37° C for 15 min and counterstained with anti-fade Vectashield mounting medium (Vector Laboratories, Burlingame, CA, USA) along with DAPI for Laser confocal microscopy by using LSM 510 microscope (Carl Zeiss GmbH, Germany).

### Real-time quantitative PCR

Total RNA from AC and AMC animals, as well as Scr and ShMUC4 of LS-180 and HCT-8 cell lines were extracted by using RNeasy Mini Kit (Qiagen, Germantown, MD, USA) and 1 ug of total RNA was reverse transcribed to cDNA by using 5x iScript™ RT supermix (Bio-rad, USA) according to the manufacturer’s protocol. After cDNA conversion, quantitative RT-PCR was carried out on Bio-rad CFX Connect™ Real-Time system by using SYBR Green master mix. The PCR program contains 95° C for 5 min; 40 cycles of 95° C for 30 sec, 58° C for 30 sec, and 72° C for 30 sec; and 72° C for 5 min. To obtain the Δct value, the expression of genes was normalized to GAPDH and β-actin and calculated 2^- (average Δct) [70, 71]. The list of primers used in the present study are listed in Table 1.

**Table 1:** Real time PCR primers used in this study.

**Genes**	**Forward primer (5’-3’)**	**Reverse primer (5’-3’)**
**Mouse primers**		
*Muc13*	GGACCCAGGCAAATGACAATA	CCCTGCTTTCCTACCAACTAAC
*Muc5ac*	CTGTAACACCCAGTGTCCTAAG	AGGCTGGTAGAAGTAGGTAGAA
*Muc16*	CCTCCTGAACCACAGAACATAA	CTGGATGGACAACTTTGGTAGA
*Reg3b*	AATGGAGGTGGATGGGAATG	CCACAGAAAGCACGGTCTAA
*Reg3γ*	CTTCCTGTCCTCCATGATCAAA	CCACCTCTGTTGGGTTCATAG
*Saa3*	AGCCAAAGATGGGTCCAGTT	TCAGAGTAGGCTCGCCACAT
*Tnfα*	CTACCTTGTTGCCTCCTCTTT	GAGCAGAGGTTCAGTGATGTAG
*Il6*	AGTTGCCTTCTTGGGACTGA	TCCACGATTTCCCAGAGAAC
*Cxcl2*	GACAGAAGTCATAGCCACTCTC	GCCTTGCCTTTGTTCAGTATC
*Ccl2*	TGTGCTGACCCCAAGAAGG	GGTGGTTGTGGAAAAGGTAGTG
*Cxcl1*	CACCCAAACCGAAGTCATAGC	GAAGCCAGCGTTCACCAGA
*Il1*	GGGCTGGACTGTTTCTAATGC	CTTGTGACCCTGAGCGACC
*Ctnnb1*	GACACCTCCCAAGTCCTTTATG	CTGAGCCCTAGTCATTGCATAC
*Ccnd1*	CAACAGGTTGTAGGGCTGGT	GGTAATGCCATCATGGTTCC
*Cd44*	TGGATCCGAATTAGCTGGAC	AGCTTTTTCTTCTGCCCACA
*Gapdh*	ATCAAGAAGGTGGTGAAGCA	AGACAACCTGGTCCTCAGTGT
**Human primers**		
*CTNNB1*	GAAACGGCTTTCAGTTGAGC	CTGGCCATATCCACCAGAGT
*CCND1*	GAGGAAGAGGAGGAGGAGGA	GAGATGGAAGGGGGAAAGAG
*CD44*	AAGGTGGAGCAAACACAACC	AGCTTTTTCTTCTGCCCACA
*ACTB*	CACCAACTGGGACGACAT	ACA GCC TGG ATA GCA ACG

### Sub-cellular fractionation

For cytoplasmic and nuclear fractionation, the LS-180 cell line was rinsed with ice-cold PBS and incubated in the presence of cytoplasmic-extraction buffer [10 mM HEPES (pH 7.4), 10 mM KCl, 0.2% NP-40, 0.1 mM EDTA, 10% glycerol, 1.5 mM MgCl_2_, supplemented with protease inhibitor cocktail, 1 mM DTT, 1 mM PMSF, 5 mM Na_3_VO_4_, and 5mM NaF] for 1 hr. The cytoplasmic fraction (supernatant) was collected after centrifugation at 16000 x*g* for 20 min. Next, pellet was washed with PBS by multiple centrifugations (3x5’) at 16000 x*g*, after that incubated with nuclear extraction buffer [20 mM HEPES (pH 7.6), 420 mM NaCl, 1 mM EDTA, 20% glycerol, 1.5 mM MgCl_2_, 1 mM DTT, 1 mM PMSF, 5 mM Na3VO4, 5 mM NaF] for 1 hr on ice, sonicated at 60% amplitude for 10s and again centrifugation at 16000 x*g* for 15 min and the resultant supernatant was a nuclear extract.

### Western blot analysis

Colon tissues from AC and AMC mice or CRC cell lines were lysed in RIPA lysis buffer along with a protease inhibitor cocktail (Roche Diagnostics, Mannheim, Germany). Equal amounts of total protein were loaded and separated by 10-12% sodium dodecyl sulfate–polyacrylamide gel electrophoresis and transferred onto polyvinylidene difluoride membranes. Due to its high molecular weight, MUC4, MUC5AC and MUC1 were resolved in 2% SDS-agarose gel. After blotting, membranes were blocked for 1 hr in 5% non-fat dry milk containing phosphate-buffered saline and 0.1% Tween 20 (v/v) and incubated with indicated primary antibodies overnight at 4° C, followed by incubation for 1 hr with respective secondary antibodies and protein bands were detected with a chemiluminescence reagent (GE Healthcare Bio-Sciences, Pittsburgh, PA, USA).

### Statistical analysis

All statistical analysis was performed by using Student’s t-test, and the data were analyzed using GraphPad Prism Software 7.0 (San Diego, CA, USA). All the data are presented as mean ± standard errors of the mean. *p*<0.05 was considered statistically significant.

## Supplementary Material

Supplementary Figures
