# Supplementary material for: Depletion of transmembrane mucin 4 (Muc4) alters intestinal homeostasis in a genetically engineered mouse model of colorectal cancer
Source: Aging (Albany NY). 2022 Mar 7;14(5):2025–46. doi: 10.18632/aging.203935 (PMC8954958; doi:10.18632/aging.203935)
Supplement: Supplementary Figures [file aging-14-203935-s001.pdf]

SUPPLEMENTARY FIGURES

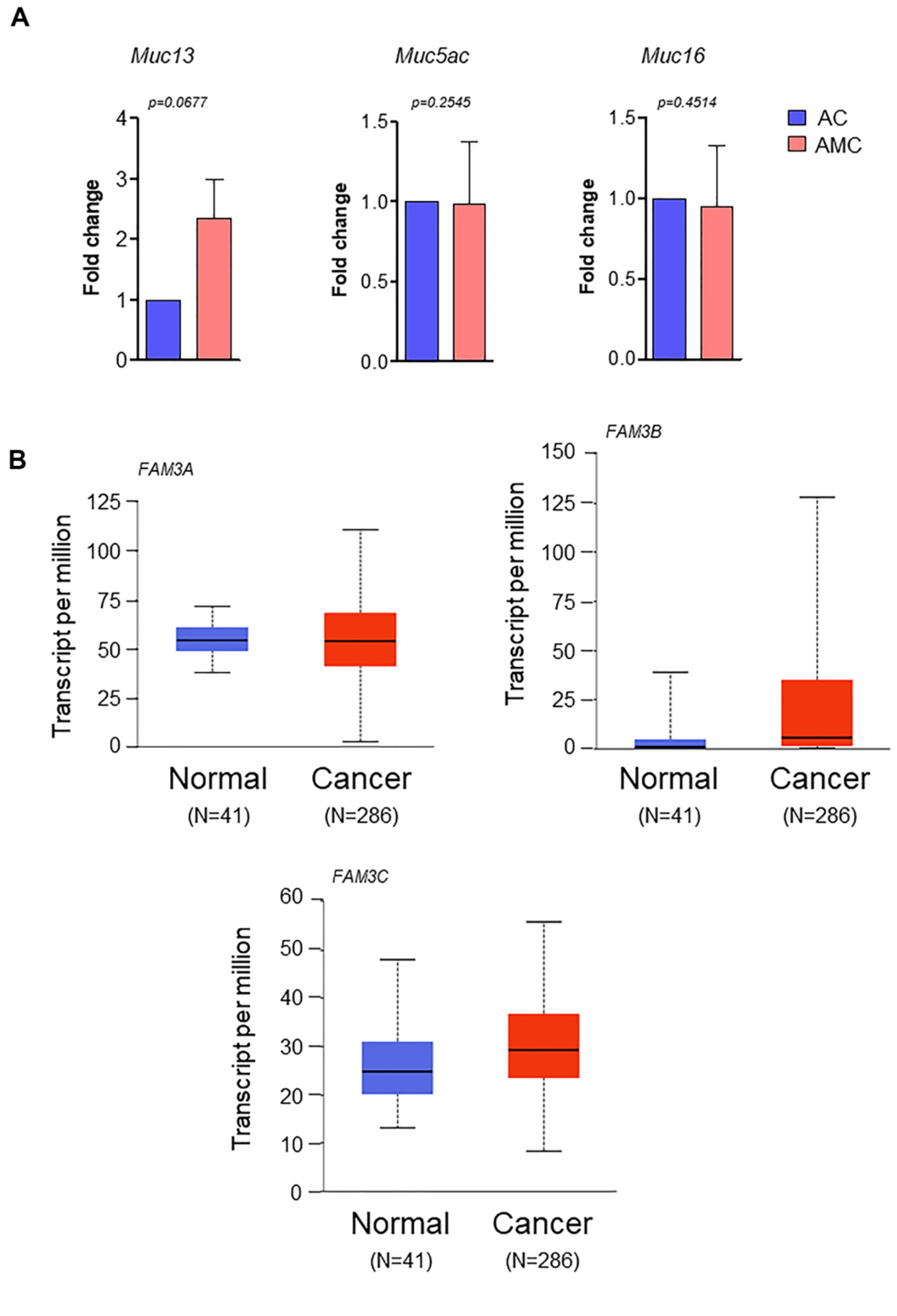

Supplementary Figure 1. The absence of Muc4 alters other mucins and FAM3 expressions. (**A**) mRNA expression levels of transmembrane (*Muc13*, *Muc16*) and secreted mucin (*Muc5ac*) in the colons of AC and AMC mice measured by real-time PCR. n = 4 per group. (**B**) TCGA-COAD expression analysis of FAM family members (FAM3A, FAM3B, and FAM3C).

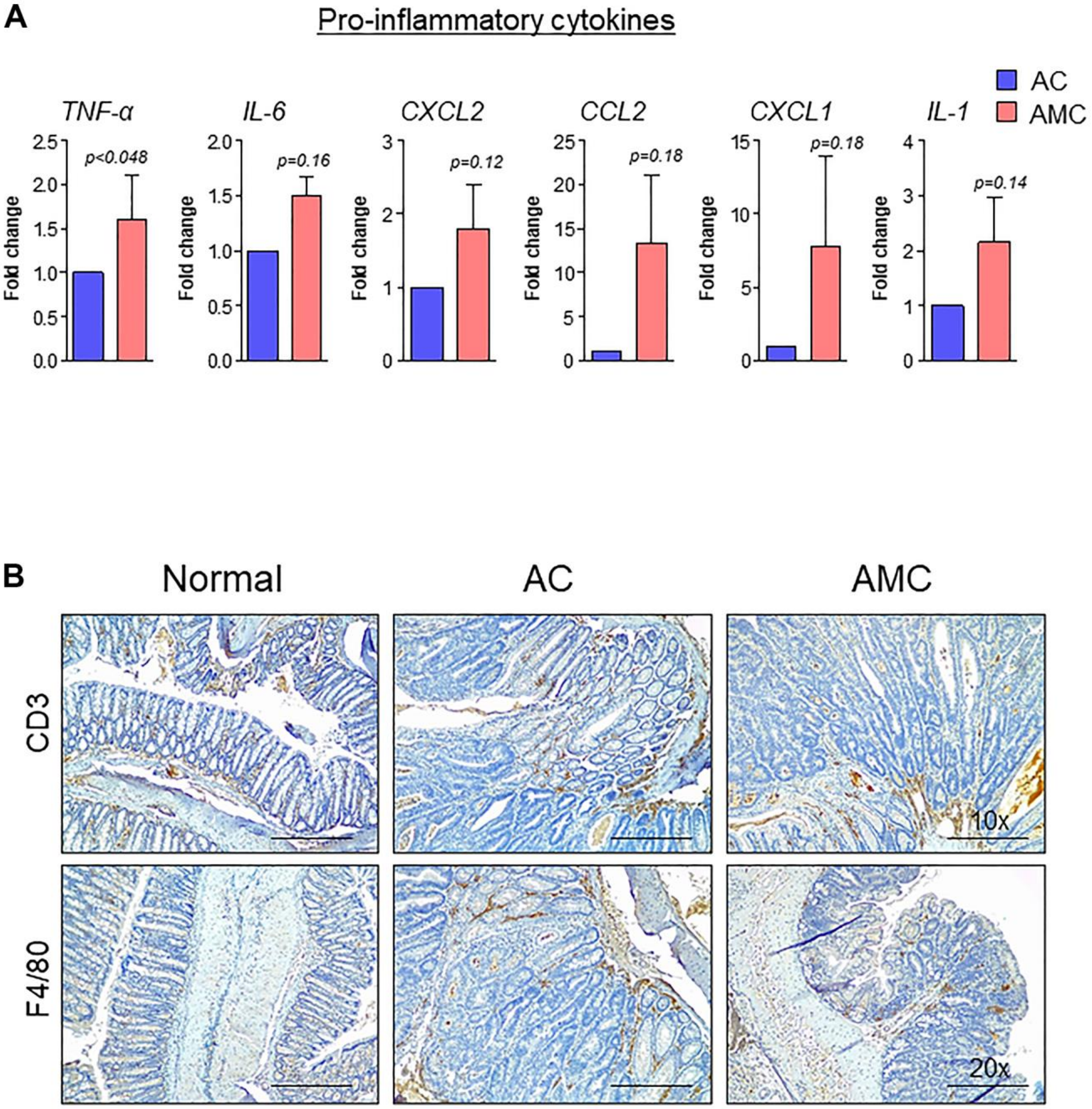

Supplementary Figure 2. Loss of Muc4 showed changes in the expression of cytokines and inflammatory markers. (**A**) mRNA expression levels of proinflammatory cytokines (*Tnfa, IL-6, Cxcl2, Ccl2, Cxcl1 and IL-1*) in the colons of AC and AMC mice measured by real-time PCR. n = 3-4 per group. (**B**) Immunohistochemical staining of immune filtration markers ($CD3^{+}$ and F4/80) in normal, AC, and AMC mice. n = 6 (AC and AMC) and n=3 for normal group.

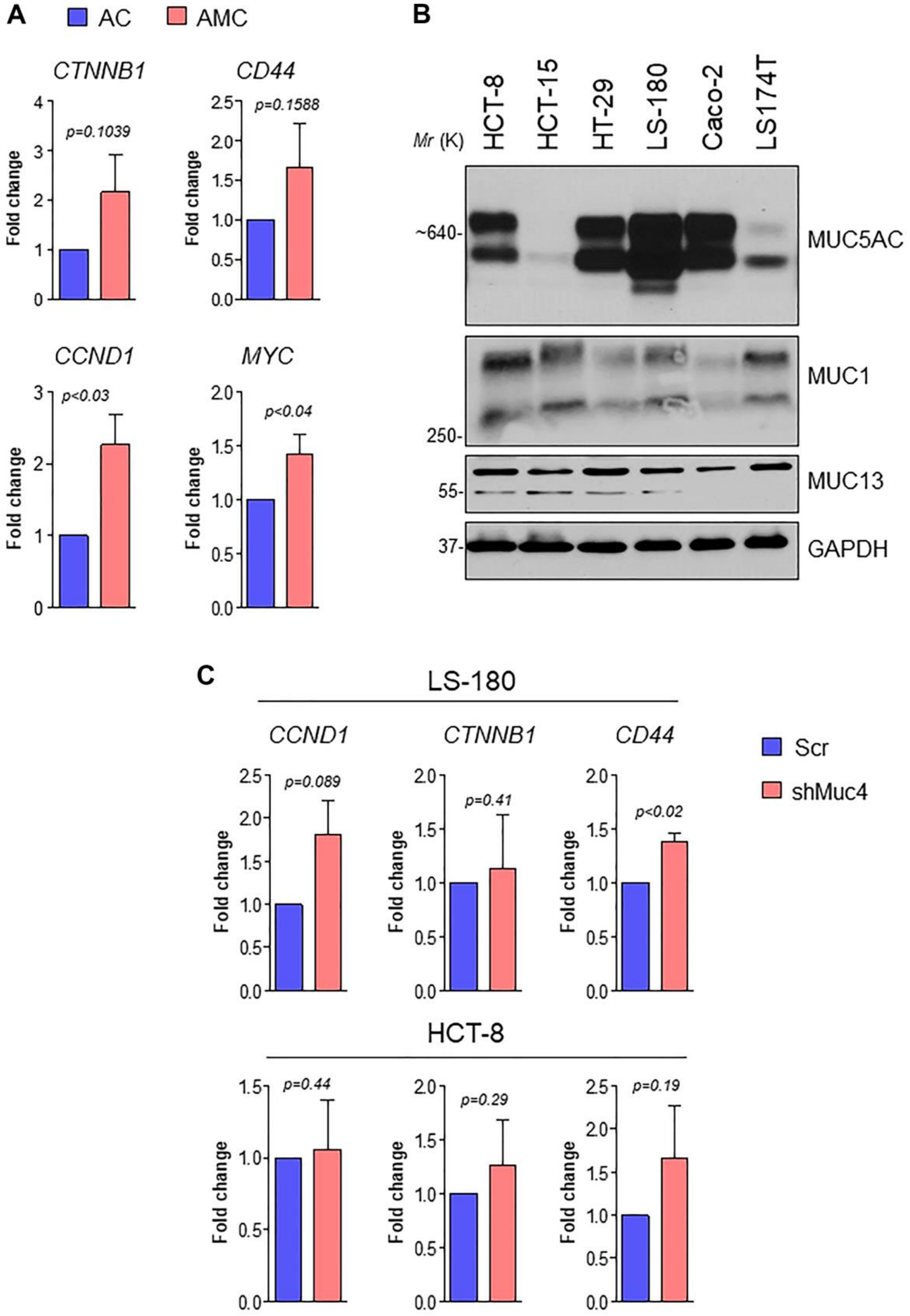

Supplementary Figure 3. The absence of Muc4 mediates upregulation of β-catenin and its target genes (**A**) mRNA expression levels of *Ctnnb1, Cd44, Ccnd1, and Myc* in the colons of AC and AMC mice measured by real-time PCR. n = 4 per group. (**B**) Screening different types of mucins (MUC5AC, MUC1 and MUC13) in a panel of CRC cell lines by western blot. (**C**) mRNA expression levels of *CCND1, CTNNB1, and CD44* in Scr and MUC4-KD CRC cell lines (LS-180 and HCT-8) measured by real-time PCR. n = 3 per group.
